# Cryonics in the Courtroom: Which Interests? Whose Interests?

**DOI:** 10.1093/medlaw/fwx045

**Published:** 2017-10-25

**Authors:** Richard Huxtable

**Affiliations:** Centre for Ethics in Medicine, School of Social and Community Medicine, University of Bristol, UK

**Keywords:** Autonomy, Best Interests, Capacity, Cryonics, Identity, Public interest

## Abstract

In an apparent international first, the High Court has allowed a terminally ill 14-year-old to be cryopreserved after her death. The patient, JS, requested this, as she hoped one day to be reanimated and cured. Jackson J focused on the welfare (or best interests) of JS as she approached the end of her life and particularly on her (apparently) competent wish to be cryopreserved. I consider the interests involved in a decision to undergo cryonics, specifically exploring which interests and whose interests are engaged. Starting with autonomy interests, the judgment implicitly supported a relational account of autonomy, but was dominated by a subjective interpretation of autonomy, which prioritized JS’s wishes. Questions nevertheless arise about whether the dying person is entitled to legislate for the reanimated person he or she might become. Temporal concerns also feature when we interpret welfare in terms of happiness, because the dying person and the (potential) future reanimated person might have different interests at different times. Finally, I widen the analysis to accommodate the interests of others, by exploring whether cryonics is in, or contrary to, the public interest. Utilizing different accounts of the public interest, I argue that the case for cryonics is not entirely made out. These observations on autonomy, happiness and the public interest combine to suggest that, although there may not be a decisive case for denying a wish like JS’s, there is a case for caution, at least while we seek to clarify and resolve the different interests in issue.

## I. INTRODUCTION

I have been asked to explain why I want this unusual thing done. I’m only 14 years old and I don’t want to die, but I know I am going to. I think being cryo-preserved gives me a chance to be cured and woken up, even in hundreds of years’ time. I don’t want to be buried underground. I want to live and live longer and I think that in the future they might find a cure for my cancer and wake me up. I want to have this chance. This is my wish.^1^

These are the words of JS, a terminally ill 14-year-old who sought—and obtained—approval from the High Court to have her body cryopreserved after her death, in the hope that she might one day be reanimated. This was apparently the first time that any court, internationally, has been asked to approve such a request.^2^ Cryopreservation and reanimation of various tissues is already possible,^3^ but reanimation of the sort sought by JS is not (yet?) possible. This type of cryopreservation is known as ‘cryonics’,^4^ a term first used in the mid-1960s, around which time the cryopreservation of cadavers also began.^5^ The movement was spearheaded by American physicist Robert Ettinger, whose book *The Prospect of Immortality* was initially published in 1962.^6^

There currently appear to be five organizations that provide a cryonics service: the Cryonics Institute,^7^ to which JS’s body was reportedly transferred,^8^ Alcor,^9^ Oregon Cryonics,^10^ Trans Times,^11^ and KrioRus.^12^ The first four of these are based in the USA, the latter is based in Russia, and a new company plans to open a facility in Australia in 2017.^13^ There are also various organizations worldwide dedicated to the cryonic cause,^14^ including research groups, organizations that help to facilitate cryopreservation, and, in the case of the Society for Venturism, a church, whose members ‘believe that we have a religious right to object to autopsy, which would interfere with our ability to go into cryopreservation in a timely fashion upon our legal deaths’.^15^

Cryonics involves cooling the body (or only the head) to very low temperatures. The Cryonics Institute explains that its procedure has four phases, from pre-treatment, to initial cool down and transportation, to washout and perfusion, before further cooling and storage.^16^ They, like the other companies, outline the costs entailed, explaining that many prospective cryons choose to arrange payment through life insurance.^17^ The organizations readily admit that the science of possible reanimation and cure is not yet perfected, with customers merely being ‘afforded the opportunity to be preserved at cryogenic temperatures in hope that future medical technology may be able to someday revive and restore them to full health’.^18^

Jackson J recognized that JS’s case invited reflection on a ‘speculative and controversial’^19^ procedure, but he focused his decision on JS’s current welfare and, in particular, her wishes. This was a difficult decision, humanely decided.^20^ In this article, I will nevertheless critically explore the interests in issue in such a case, with a particular focus on *which* interests and *whose* interests are at stake. Although I will raise more questions than I answer, I hope to show that there are significant challenges associated with not only balancing the current and future interests of individuals like JS, but also balancing the interests of JS (at whatever time) against those of the wider community (again, at whatever time).

I will first work through the autonomy interests. Some objective, relational considerations featured in the ruling, but Jackson J essentially followed the dominant legal line in deploying a subjective account of autonomy, which looked to—and respected—JS’s particular wishes. This appears to be appropriate, but I nevertheless query whether the dying JS has the authority to legislate for the future JS that she might become, both as a ‘cryon’,^21^ and (if her hopes are realized) as a reanimated individual. Temporal concerns also arise when I analyse JS’s welfare in terms of her happiness. Here, for example, I explore the potential happiness that might be enjoyed by a reanimated JS, but consider how this might impose some unhappy costs on her life—and death—prior to cryopreservation.

I then expand the analysis to include the public interest, and thus the interests of the community at large. Such considerations also feature in law, although—as was the case in this ruling—they are not always fully articulated or explicated. Drawing on different accounts of the public interest, I argue that the case for cryonics is not entirely made out, as it appears (unjustly) to be the preserve of the wealthy, has an adverse environmental impact, and risks uncertainty about the definition of death (and what follows thereafter). Science should be free to flourish but we might ask whether, in effectively endorsing JS’s speculative hope, the court in this case has overstepped a public interest line. At best, this ruling offered some hope and comfort to a dying teenager; at worst, it offered false hope to JS and cold comfort, not only to JS, but perhaps also to the wider society. These observations combine to suggest that there may not be a decisive case for prohibition and thus the denial of a wish like JS’s, but there is a case for caution, at least while we seek to clarify and resolve the different interests in play. Before advancing these arguments, I will set the scene, by outlining the decision in *Re JS*.

## II. CRYONICS IN THE COURTROOM

14-year-old JS had been diagnosed with a rare form of cancer in 2015 and, by August 2016, was receiving palliative end-of-life care. Following some months of research, she formed a wish for cryonics: ‘I want to live and live longer and I think that in the future they might find a cure for my cancer and wake me up. I want to have this chance. This is my wish.’^22^ Her mother supported her request and her grandparents secured the £37,000 needed to cover the procedure. JS’s estranged father (with whom JS had not had contact since 2008) ultimately agreed to offer no objection, although he wanted he and his family to be permitted to view JS’s body after her death, to avoid contact with JS’s mother, and to avoid any financial liability.

The High Court was approached to resolve the dispute between JS’s parents and determine the lawfulness of JS’s wish. Jackson J found that he had the power to resolve these matters, and he essentially ruled in favour of JS and her mother. As required by the Children Act 1989, JS’s welfare was Jackson J’s paramount consideration and, although he evidently felt some discomfort with JS’s choice, for him ‘the predominant features are JS’s wishes and feelings and her acute emotional needs’.^23^ A specific issue order was made under the Children Act 1989, which allowed JS’s mother to continue to make arrangements for the preservation of JS’s body. Via a prospective order, she was also made the sole administrator of JS’s estate, with sole authority to make arrangements for the disposal and viewing of JS’s body. JS’s father was assured he would not be financially liable, but he was prevented (by an injunction *in personam*) from seeking to administer JS’s estate or otherwise interfering with the arrangements made by JS’s mother.

The central issues resolved, Jackson J nevertheless appreciated that this was a novel case involving a ‘speculative and controversial’ science,^24^ which was not wholly covered by the existing law, such as is as set down in the Human Tissue Act 2004. Recognizing that the case might ‘suggest the need for proper regulation of cryonic preservation in this country if it is to happen in future’,^25^ he ordered the disclosure of papers to the Human Tissue Authority. Initial preservation of JS’s body was reportedly arranged by Cryonics UK, before it was then transported to the Cryonics Institute in the USA.^26^ Sadly, as Jackson J relayed in a post-script, JS’s mother was said to have been ‘preoccupied with the post-mortem arrangements at the expense of being fully available to JS’ on her last day, while the voluntary organization was apparently ‘under-equipped and disorganised, resulting in pressure being placed on the hospital to allow procedures that had not been agreed’.^27^

Jackson J suspected that ‘this application is the only one of its kind to have come before the courts in this country, and probably anywhere else’.^28^ Cryonics has made it into court elsewhere in the past, albeit in rather different circumstances: for example, in an American case in 1981, a jury awarded ‘$928,594.00 for breach of contract and fraud regarding a bankrupt cryotorium’s failure to provide the continuous suspension of two individuals’, while in 1992 a Californian court rejected a plea to allow pre-mortem cryonic suspension.^29^ As Jackson J noted, *Re JS* ‘is an example of the new questions that science poses to the law, perhaps most of all to family law’.^30^ In this case, the dominant consideration from family law was that of the welfare or ‘best interests’ of JS.^31^ The Californian court had been similarly alert to the interests of the individual, but it notably also considered the wider public interests involved. In what follows, I seek to explore the different sets of interests engaged, specifically investigating which interests and whose interests are in issue in JS’s case. I start with JS’s autonomy, which was central to Jackson J’s reasoning.

## III. IN THE INTERESTS OF JS’S AUTONOMY?

According to the Children Act 1989, JS’s welfare had to be Jackson J’s ‘paramount concern’ when deciding whether or not to grant a specific issue order that would empower JS’s mother to make arrangements for JS’s cryopreservation.^32^ The judge opined that ‘the predominant features’ of JS’s welfare were ‘JS’s wishes and feelings and her acute emotional needs’.^33^ Here, Jackson J gestures towards two of the three dominant approaches to welfare taken in axiology (value theory), which Parfit introduces as follows:

What would be best for someone, or would be most in this person’s interests, or would make this person’s life go, for him, as well as possible? Answers to this question I call theories about self-interest. There are three kinds of theory. On *Hedonistic Theories*, what would be best for someone is what would make his life happiest. On *Desire-Fulfilment Theories*, what would be best for someone is what, throughout his life, would best fulfil his desires. On *Objective List Theories*, certain things are good or bad for us, whether or not we want to have the good things, or to avoid the bad things.^34^

When referring to JS’s ‘wishes and feelings’, Jackson J is implicitly appealing to desire-fulfilment theories, which effectively align with accounts of respect for autonomy (self-rule). Although accounts of autonomy abound, healthcare law appears most inclined towards *subjective*, rather than *objective*, understandings of the concept.

On a subjective account of autonomy, a person’s decision warrants respect if it expresses either their ‘current’ (i.e. even fleeting or uncritically held) desires or their ‘best’ desires, where the latter refers to those desires that align with the individual patient’s value system, whatever those values happen to be.^35^ On an objective account of autonomy, only an ‘ideal’ set of desires warrant respect i.e. the person must take a ‘responsible’ decision, which aligns with ‘a purportedly objective system of ideals’.^36^ On such an account, the content of the person’s decision can be scrutinized: only a decision with the ‘right’ content should be respected.

Healthcare law generally favours a subjective approach to autonomy, particularly (but not exclusively) where adult patients are concerned.^37^ Capacity or competence operates as a gatekeeper to autonomy: if the patient has capacity in the relevant sense, then his or her individual decision generally warrants respect.^38^ In its reports preceding the Mental Capacity Act 2005, the Law Commission contemplated three broad approaches to capacity: the ‘status’, ‘outcome’, and ‘functional’ approaches.^39^ ‘Status’-based approaches do feature in the law, at least in relation to minors: for example, the test of competence that applies to under-16s differs from that applicable to over-16s,^40^ and a refusal of treatment by a competent minor of any age lacks the legal force of a refusal issued by a competent adult.^41^ Yet, the law generally favours ‘functional’ approaches to capacity: both adults and children will have (at least some of) their decisions respected, if they are able to understand the nature and effect of those decisions. An ‘outcome’ approach, meanwhile, finds no explicit support in law.^42^ According to the Law Commission, this approach

focuses on the final content of an individual’s decision. Any decision which is inconsistent with conventional values, or with which the assessor disagrees, may be classified as incompetent. This penalises individuality and demands conformity at the expense of personal autonomy.^43^

The law’s preference for a functional—rather than an outcome-based or (entirely) status-based—approach to capacity indicates that the law generally supports a subjective, as opposed to an objective, account of patient autonomy. That preference is also increasingly evident in the courts’ dealings with patients who lack or lose capacity: as Lady Hale noted in *Aintree*, ‘The purpose of the best interests test is to consider matters from the patient’s point of view’.^44^

Given the legal framework, Jackson J unsurprisingly took a subjective approach to JS’s autonomy. This orientation is evident in his reference to the wishes and feelings of JS, rather than those of some ‘objective’ other. However, objective considerations can still be detected in the ruling. We will start with these, before exploring Jackson J’s subjective approach to JS’s wishes, and then querying whether these wishes should have proven so influential.

### A. Autonomy as Objective

Although Jackson J essentially took a subjective approach to JS’s autonomy, objective considerations can be glimpsed in the ruling, and perhaps unsurprisingly so, given the law’s (aforementioned) reluctance to empower minors to make decisions that might be contrary to their welfare. The content and outcome of JS’s decision is (to echo the Law Commission) certainly unconventional and, as Jackson J notes, ‘all the professionals feel deep unease about it’.^45^ Jackson J seems to share the doctors’ doubts that JS is making the ‘right’ decision. He also indicates that he understands JS’s ‘father’s misgivings’ and emphasizes that ‘The court is *not* approving or encouraging cryonics, still less ordering that JS’s body should be cryonically preserved’.^46^ Like other judges before him,^47^ Jackson J appears not to like the patient’s choice—but he nevertheless confirms that it is indeed her choice and that it merits respect.^48^

Perhaps, however, Jackson J can be somewhat reassured, since respecting JS’s decision does at least help to promote one objective good, since it honours the longstanding bond between JS and her mother. Engaging in deep relationships is one of the objective goods cited in discussions of welfare.^49^ Autonomy–at least as subjectively conceived—can appear to be at odds with the maintenance of such relationships. Indeed, it is striking that English law is further entrenching a subjective account of autonomy, at a time when the concept is coming under increasing attack in bioethics. Subjective accounts of autonomy are said to be misconceived, over-inflated, and neglectful of social values like solidarity.^50^ As Lanre-Abass puts it:

too much stress on autonomy can lead to an isolation of the subject and even distort people’s understanding of the way individual decisions are embedded in a web of relationships and familial values. Also, stressing individual autonomy to the exclusion of other values can do real harm to families.^51^

But Jackson J’s decision arguably avoids doing such harm: mother and daughter are united here, as indeed they had been for many years.^52^ In effect, Jackson J’s ruling appears to avoid the atomization feared by those who prefer a relational ethic to govern, and this might be considered objectively worthwhile.

### B. Autonomy as Subjective

Jackson J’s ruling is therefore amenable to an objective reading, since it honours the bond between JS and her mother, but the better reading is that he understood and applied a subjective account of autonomy. As a first step, JS’s competence had to be confirmed. For a 14-year-old like JS, competence is to be assessed by reference to the decision in *Gillick*, in which Lord Scarman referred to ‘the child’s right to make his own decisions when he reaches a sufficient understanding and intelligence to be capable of making up his own mind on the matter requiring decision’.^53^ Jackson J confirmed ‘that JS has the capacity to bring this application’,^54^ elsewhere stating ‘that JS is a child, albeit a legally competent one’.^55^ Although questions arise here,^56^ Jackson J makes clear that he believes JS to have the requisite functional competence:

She is described by her experienced solicitor as a bright, intelligent young person who is able to articulate strongly held views on her current situation. Her social worker says that she has pursued her investigations with determination, even though a number of people have tried to dissuade her, and that she has not been coerced or steered by her family or anyone else.^57^

In addition to indicating her competence, these observations imply that JS’s decision is one that she would endorse on reflection, as it is consistent with her values – in other words, with her (subjective) ‘best’ desires, as opposed to merely her ‘current’ desires.

However, at least two objections can be levelled at the idea that JS’s decision articulates her best desires – although both objections can be met. First, there is the problem of timing. We are told that JS pursued her investigations ‘over recent months’,^58^ and hence during the period that she was dying, with all the anguish and suffering that this might sadly have entailed. There is also her young age to consider: can a 14-year-old form and hold the relevant ‘best’ desires? In answer to this objection, however, we should accept not only that adults’ values can change,^59^ but also that there is evidence to suggest that young people–particularly those who endure longstanding or terminal illness – can form autonomous treatment preferences.^60^

Secondly, there is the problem of information. Jackson J seemed satisfied that JS had the ‘sufficient understanding and intelligence’ to which Lord Scarman referred. But understanding requires information. How can JS have been sufficiently informed? Presumably JS’s research revealed what is known about cryonics, in terms of its mechanics, costs, and the fact that the science cannot yet deliver on its potential promises of a future cure and reanimation.^61^ But cryonics also involves known unknowns (and, no doubt, unknown unknowns). If the procedure does ultimately deliver, then what future can JS anticipate?

In response to these queries, we should first accept that such questions can be asked of any and every decision to undergo cryopreservation, whatever the age or health of the potential cryon. Moreover, they might well be asked of any and every decision ever contemplated, by anyone. In short, it is impossible for anyone to be *fully* informed about any decision: one can only know what a decision entails after having taken that decision. And every decision is irreversible, if only in temporal terms: one cannot time-travel back to the point of decision, in order to take a different decision.^62^ Absolute informedness is impossible to attain, and if we were to raise the bar on the level of information that is required before a decision can be considered autonomous, we are likely to find ourselves deprived of numerous opportunities to decide. The law already permits people to, in effect, choose death, through the refusal of life-sustaining treatment.^63^ For many, death is unknowable: one might be informed about what continued life is likely to involve, but one cannot be similarly informed about death. Despite this, the law protects these choices, and finds that they can be autonomously made. What Jackson J achieves is therefore arguably consistent with this position: JS had the relevant *available* information, and the necessary intelligence, so her choice should similarly be respected.

Jackson J therefore honoured JS’s subjective, autonomous wishes, and perhaps with good reason. Judging by previous cases, an objective approach was arguably available to the court, which could have enabled Jackson J not to accede to JS’s wish. Jackson J could, for example, have seized upon JS’s statement ‘I don’t want to be buried underground’,^64^ and taken this as evidence of her immaturity and incompetence.^65^ That he did not do so might signal that English law is warming to adolescent autonomy. We might query whether Jackson J’s determination ‘to remove the disadvantage that JS is under as result of her age’ might come to extend to other contexts,^66^ such as the refusal of life-saving treatment, a situation in which minors currently do not enjoy the rights available to adults.^67^ We might even ponder whether Jackson J has opened the door to patients’ demands. At present, no patient can make a demand for treatment and expect that demand to be met, no matter how fanciful or ‘futile’ it appears.^68^ Admittedly, JS’s demand primarily takes effect after her death, so hers was not strictly a demand for ‘treatment’. Nevertheless, in its effect, Jackson J’s decision arguably aligns with others that appear to be chipping away at the prohibition on honouring patients’ demands.^69^ But, regardless of the potential legal ramifications, the central point remains: Jackson J appeared to have good reason for finding JS competent and for deciding to honour her wish.

### C. Autonomy and Identity

We can nevertheless still query whether JS’s desire *should* have carried the day. For one thing, there are other interests in play (to which I will return). Moreover, retaining the focus on JS’s autonomy, we can ask: why should an autonomous choice by the living JS bind the deceased JS—and any future JS she might become, if reanimation proves possible? Had JS lived and died ‘naturally’,^70^ then, leaving aside her prenatal existence, there would be two significant time-intervals to consider: the living, but ultimately terminally ill, JS (*living-JS*) and the deceased JS (*deceased-JS*). But JS sought a different life-course, which has three or four significant time intervals: the living, but terminally ill, JS (*living-JS*); the deceased but cryopreserved JS (*cryon-JS*); and the reanimated JS who, let us assume, has been or will shortly be cured of her cancer (*reanimated-JS*); *reanimated-JS* might later die (becoming *deceased-JS*), unless the science has developed to enable life to be sustained indefinitely. What entitles *living-JS* to bind the future JS’s?

This is a question about precedent autonomy. In effect, JS has made a ‘living will’ and, strictly speaking, hers might be the first such direction to have received formal legal approval. That term, coined by Kutner in 1969, has fallen out of favour as a label for advance medical directives, since a will only strictly applies after death.^71^ But the term seems right in this specific context: JS is living and has made a will regarding future medical intervention that is to apply after her death. Precedent autonomy is a challenging enough notion in the contexts in which it is usually aired, such as when a capacitous individual seeks to make an advance treatment decision for the incapacitous individual he or she might become.^72^ The major philosophical challenge is one of personal identity: is the drafter of the directive the same individual to whom it will apply and, if not, what is the source of his or her authority for binding the future entity?

Although subtler positions are available,^73^ ‘animalist’ and psychological accounts of personal identity tend to dominate. In the former camp, animalists point to identity enduring over time by virtue of a persisting body; in the latter, psychological connections are emphasized. Psychological accounts will struggle with advance decision-making, but animalist accounts can entitle me to make provision for a future in which I have mild dementia (in which, admittedly, I retain some psychological connection to my pre-demented self), advanced dementia (in which the psychological connections appear much weaker), or am in a permanent vegetative state (in which I have apparently lost higher brain function).^74^

However, in the case of cryonics, even an animalist account might struggle to find the relevant continuity. In the previous examples, the body persisted as a living entity. In the future JS hoped for, she would be dead—although she could possibly (but only possibly) thereafter be brought (back) to life. An animalist might therefore say that there are qualitative differences between each of these three JS’s, such that there might be three different JS’s to consider: the first is living, with cancer; the second is replete with cryopreservatives; the third is (presumably) cured of the cancer. Someone more inclined to psychological connectedness as being constitutive of identity would certainly perceive a difference between *living-JS* and *cryon-JS*, and might be tempted to argue that *reanimated-JS* will be qualitatively (psychologically) affected by the journey she has undergone.^75^

But these objections might not succeed. First, it could be countered that we already empower people to extend their wishes beyond their deaths, through the ability to make wills and indicate their wishes as to the disposal of their remains. Moreover, secondly, Shaw and Moen, who have independently written in support of decisions to undergo cryonics, imply that JS does have the requisite authority, since *reanimated-JS* is identifiably the same individual as *living-JS*. Shaw analogizes cryopreservation with the provision of life-sustaining treatment: ‘in essence, successful cryonics would be a form of life-support that delays, rather than returns the user from, death’.^76^ What he assumes, but does not explicitly say, is that the identifiable individual known as JS will persist over time, both as a physical and psychological entity, albeit with a cryonic pause (reflected in the term ‘cryonic suspension’).

Moen tackles the identity point head-on, by analogy with those who undergo neurosurgery or are revived after having drowned in cold water:

Since we know that cryopreservation can render fine biological materials (including neurons) intact, chances are good that it can also render intact the neural structures that encode personality, thoughts and memories. Even if we think that neither particles nor patterns of particles are directly relevant to personal identity (perhaps we hold a psychological continuity theory or we believe in a soul), it is unclear what more, on a physical, and thus medically relevant, level we can require for survival than the same particles being arranged in the same pattern.^77^

As such, for Moen and Shaw, an entity that is identifiable as JS can persist over time, so, by extension, the living JS has the right to make decisions for the future cryopreserved JS. Perhaps they have a point, otherwise we would have to concede, for example, that anyone who has had cancer and successfully undergone treatment has become a different person, either by virtue of the physical changes effected by the treatment or by virtue of the experiences they have had. However, that only accounts for the *living-JS* and *reanimated-JS*: *cryon-JS* remains to be understood. But maybe there is no problem here either, otherwise those who have been successfully resuscitated would also have to be considered different people.

Whether JS has the authority to bind the future JS she might become nevertheless remains a moot point, which I cannot seek to resolve here. Hopefully, however, I have done enough to suggest that there are considerable philosophical complexities surrounding Jackson J’s reasoning, which concern *whose* interests—and thus choices—the court should have in view. The temporal point recurs, albeit in a different way, when we move to another ethical school of thought that features in Jackson J’s ruling, according to which an individual’s welfare rests on his or her happiness.

## IV. IN THE INTERESTS OF JS’S HAPPINESS?

Although Jackson J placed substantial weight thereon, he did not only rely on JS’s (subjective) wishes and feelings in reaching his decision, as he also had regard for ‘her acute emotional needs’.^78^ As such, his reasoning was also informed by the hedonistic theories to which Parfit referred, according to which a person’s welfare is determined by his or her happiness. For its part, the hospital reportedly saw JS’s happiness as *contingent* on her desires being honoured. As Jackson J explained, ‘the hospital is willing to do what it properly can to cooperate for the sake of JS, because the prospect of her wishes being followed will reduce her agitation and distress about her impending death’.^79^ On this account, JS’s desires inform, but are subordinate to, her happiness. Whether or not Jackson J conceived of JS’s happiness in quite this way, he certainly shared the hospital’s inclination to address JS’s ‘present distress’ and the need to consider ‘JS’s welfare during life’.^80^

The temporal emphasis in Jackson J’s decision is notable: the judge appreciates that his ruling is directed towards the future, which is precisely where JS is looking, but he strives to locate his reasoning in the here-and-now, looking to JS’s interests as her life nears its end. As with his emphasis on JS’s wishes, this is *prima facie* plausible and, indeed, humane. However, questions also arise here. Even if we are inclined to hold that one identifiable JS persists over time, we might still wonder whether her interests will vary over time and, if so, which interests at which point in time should take priority. In short, Jackson J focuses upon the interests of *living-JS* but, if JS’s hopes for reanimation come to be realized, then the interests of *cryon-JS* and *reanimated-JS* merit consideration.

Shaw and Moen implicitly recognize that interests can differ over time, but they conclude that, on balance, a prudential and an ethical case can be made for cryonics, with each author knocking down various objections that can be levelled at the practice. Both appear most inclined to a hedonist account of welfare. Hedonism is associated with consequentialist, and specifically utilitarian, thinking, and Moen explicitly refers to the ‘utility-value’ of cryonics.^81^ Shaw’s hedonistic leanings feature in his defence of the Cryonic Wager, which is based on Pascal’s Wager. Pascal’s Wager, which is explicitly articulated in terms of ‘happiness’,^82^ suggests that the possible benefits of believing in God mean it is worth doing so or attempting to do so. Shaw adopts this form of argument to defend the Cryonic Wager: given the chance of reanimation, as opposed to the certainty of obliteration, self-interest provides a reason for undergoing cryopreservation.

So what are the happiness gains that Shaw and Moen detect, and how do these relate to each interval of JS’s persistence over time? Starting chronologically with *living-JS*, the authors believe the financial costs associated with cryonics are not prohibitive (and can be ameliorated via insurance), so prospective cryons should still be able to ‘live life to the full’.^83^ They say less about the interests of *cryon-JS* and *deceased-JS*, and perhaps understandably so, as this would require them to enter into the murky realm of posthumous interests.^84^ However, they do acknowledge a risk of reanimation not occurring, perhaps because the technology never matures, the future State has ethical objections to reanimation, the cryonics institute is bankrupted, or there is some cataclysmic event preventing reanimation. They nevertheless imply that, in such circumstances, *cryon-JS* is equivalent with *deceased-JS*, who—they further imply—has no (or no relevant) interests, precisely because she is dead. Any money spent on cryopreservation will not have been wasted because *deceased-JS* could not have used it anyway and it was better to take the chance (however small) of successful reanimation. Moen does then acknowledge the idea ‘that dying is good for us as individuals’,^85^ which implies it is better to be *deceased-JS* than *cryon-JS*. But not so, says Moen: medicine is (rightly or wrongly) founded on the denial of death and, furthermore, it is not necessarily the case that cryonics will lead to immortality.^86^

However, Moen and Shaw have most to say about the happiness of *reanimated-JS*, concluding, in Moen’s words, that we might ‘expect postcryonic life to be tolerably good’.^87^ They argue, for example, that future technology might successfully repair damaged bodies and that the reanimated individual need not be lonely as he or she could be joined by loved ones and, in any case, new relationships can be forged. They also suspect that this refugee ‘from another time’ will find a place in their new society and might be welcomed as a ‘living time capsule’.^88^ Moen cites Aristotle, who might happily fit in if he were transplanted to our time and place, as he ‘would be deeply intrigued by contemporary science, technology and philosophy, and … his life would be very much worth living’.^89^

Although they score some critical points, Moen and Shaw fail adequately to answer some relational concerns about *reanimated-JS*, which have a bearing on her future happiness. These concerns relate to her family (who will care for the reanimated person, particularly if she is a minor?), future finances (how will the reanimated person support themselves?) and fit (how comfortably will the reanimated person inhabit a future society?). Regarding the latter, it is doubtful that the time-travelling Aristotle entirely helps Moen. For a start, Aristotle might *not* want to time-travel: his renowned work in ethics suggests a marked interest in others,^90^ but it is arguable that the cryonically inclined are more likely to have a marked self-interest. And even if he did wish to travel to our time and place, Aristotle might not be a happy time-traveller, as we are likely to baulk at his attitudes to women, slaves, foreigners, and pederasty.^91^

Moreover, it is still not entirely clear why or when the happiness of the future reanimated person should outweigh the happiness of the currently living person. This brings us back to JS’s situation and the sad post-script from Jackson J. On the one hand, as the hospital noted, JS would gain some comfort as her life approached its end from knowing that her wishes would be honoured. On the other hand, however, there is evidence to suggest that her final hours might not have been as ‘happy’ as they could otherwise have been. We earlier saw how JS’s decision, as *living-JS*, at least expressed her relational ties to her mother, which presumably made some positive contribution to JS’s happiness. But Jackson J refers to evidence from the Trust, which leaves the opposite impression: ‘On JS’s last day, her mother is said to have been preoccupied with the post-mortem arrangements at the expense of being fully available to JS’.^92^ JS reportedly died peacefully, but apparently lacking her mother’s full engagement. We can only speculate, but it seems conceivable that this will have adversely affected not only JS in her final hours, but also her mother, both at that time and as she grieves thereafter.

If JS’s gamble pays off, and Shaw and Moen are right, then perhaps such suffering will have been worth it in the long-term, in view of JS’s future happiness gains. But this is where the general problem arises: what weight should be accorded to one’s present happiness, relative to one’s (potential) future happiness? This is a general problem for welfare calculations, and is one that is readily familiar from dilemmas around the treatment and care of incapacitated individuals.^93^ A nursing home resident with advanced dementia happily consumes meat at mealtimes, but their previous, capacitous self was a longstanding vegan, who would have objected; which interests should prevail?^94^ I will not seek to resolve this general problem here. I will, however, venture to suggest that the case for cryonics is not entirely made out. Admittedly, hindsight plays a part here, since the situation around JS’s demise only came to light after the judge’s decision had been made (but before the judgment was published, wherein the troubling post-script was added). It nevertheless reveals the possibility of some harm coming to *living-JS* as a result of the decision to be cryopreserved. Cryonics arrangements might improve, so future cryons could be able to avoid such problems. But the problem of balancing current against future interests will persist, as the future benefits to the reanimated person remain speculative and not all of the relevant objections can be dismissed.

## V. IN THE PUBLIC INTEREST?

In addition to the temporal dilemmas associated with the current and future interests of individual patients, questions arise about *whose* interests should be in issue, whether current or future. We have seen how there might be different JS’s to consider and the interests of JS’s family, in particular her mother, have also come into view, but there are others to consider, including the medical staff and, indeed, society at large. Decisions in medical law are empowered and constrained by such public interest considerations: the public interest may be invoked in order to promote or prohibit a particular course, in view of its assumed benefits or harms, respectively.^95^ The subjects of the relevant benefits or harms will vary: sometimes the public interest is invoked on *my* behalf, sometimes *yours*, and sometimes *ours* collectively.^96^

Even in the individual case, the judges will be mindful of the wider ramifications of their decisions, given, for example, the operation of the doctrine of precedent.^97^ Moreover, law has an expressive function, not only expressing values from society, but also expressing values to society.^98^ The precise public interest considerations the judges have in mind are seldom spelt out, although those mentioned in medico-legal cases include the interest in preserving life, preventing suicide, maintaining the integrity of the medical profession, and protecting innocent third parties.^99^ These factors were cited in the aforementioned Californian case, in which the court rejected a request for pre-mortem cryopreservation,^100^ and have also been cited in an English case, concerning a prisoner’s objection to being force-fed.^101^ Jackson J did not overtly cite such factors in his decision, but he was clearly mindful of the potential implications of his decision, having in view the interests of third parties and the integrity of the professionals caring for JS. Of course, Jackson J was required to treat JS’s welfare as his ‘paramount consideration’.^102^ Despite this emphasis, others’ interests have crept into welfare assessments,^103^ and Jackson J recognized that his ruling would have an impact beyond JS. He noted, for example, that ‘This situation gives rise to serious legal and ethical issues for the hospital trust, which has to act within the law and has duties to its other patients and to its staff’.^104^ However, he perceived no external obstacle to him ruling in JS’s favour, as the Trust, the American authorities, JS’s social worker, and the funeral directors were prepared to make the necessary arrangements.^105^

Jackson J felt able to rule in JS’s favour but the question arises whether the wider impacts of the decision are such that he should not have done so. In short, should individuals like JS be at liberty to choose cryonics or should a prohibitive public interest boundary be erected and enforced? In order to ascertain where the public interest lies, Held suggests we can look to *preponderance* (or aggregative) theories (which focus on desire-fulfilment), *common interest* theories (which promote that which we all have in common), and *unitary* theories (which favour objective accounts of welfare).^106^

### A. Preponderance Theories

*Preponderance* theories of the public interest, explains McHarg,

start from a subjective definition of interests, whereby individuals are seen as the best judges of their own interests, the most reliable evidence of which is their revealed preferences. Accordingly, the public interest has no independent content, but is discovered simply by aggregating individual interests; that which is in the interest of a preponderance of individuals is also in the public interest.^107^

Respect for autonomy may be the simplest argument in favour of permitting cryonics,^108^ but preponderance theories demand respect not for individual choices as such, but for the choices of the *majority*. The present dearth of cryons implies that the majority has made its choice. At the time of writing, there are only 352 cryons worldwide: ALCOR preserves 148 cryons,^109^ while the Cryonics Institute has 145,^110^ KrioRus has 51,^111^ Oregon Cryonics has 5,^112^ and Trans Times has 3.^113^ Even if the members of these organizations—who we might presume will later become cryons—are accounted for, we are still talking about only a few thousand cryons worldwide in the near future. According to preponderance theories, if this reluctance to be cryopreserved reveals the will of the people, then cryonics is apparently not in the public interest.^114^

This might be a stretch too far, however. First, it is not obvious that people have made autonomous decisions about cryonics; perhaps few even know that cryopreservation facilities exist. Secondly, even if people have autonomously decided not to opt for this for *themselves*, this need not mean that they wish to deny this option to *others*. Thirdly, this whole approach to the public interest risks suppressing the rights and interests of the minority:^115^

The minority view held by those wishing to undergo cryonic suspension certainly may be characterized as unpopular, but this should not mean that decedents who believe in cryonics and the prospect of reanimation do not deserve the legal right to choose what happens to their bodies upon their death.^116^

We have seen the high value placed on subjective understandings of personal autonomy and liberty, including in Jackson J’s ruling, and perhaps it is indeed questionable whether the wishes of the minority should be sacrificed in the way that preponderance theories might indicate. The most that can be said here is that the majority of people currently do not want to be cryopreserved, presumably because they are pessimistic about cryonics’ prospects. This does not tell us enough yet about whether individuals should be at liberty to make this choice.

### B. The Common Interest

An alternative way of viewing the public interest(s) at stake involves looking to the *common interest*, which encompasses those ‘interests which all members of the public have in common, hence comprising a category of interests distinct from those of particular individuals or groups’.^117^ On this account, ‘if the relevant benefits are to be provided for one person, they must inevitably be provided for all’.^118^ Here, important public interests come into view, as cryonics appears to threaten the common interest, since the (assumed) benefit is unjustly distributed and the practice jeopardizes our shared world.

First, even if we assume that cryonics does offer a benefit, it is a benefit only available to the wealthy. As an illustration, lifetime members of the Cryonics Institute can expect to pay a membership fee of $1,250, plus $28,000 for cryopreservation; standby and transfer costs then total $88,000 for such members, and there are likely to be additional costs associated with (for example) the services of funeral directors.^119^ Cheaper options are available (for example, preservation of only one’s head)^120^ and costs can be spread through insurance.^121^ However, one can still object that not everyone—even in the developed world—will be capable of meeting the necessary insurance premiums.

State provision for all would appear to provide an answer to this objection. The case for such provision *might* mount, at least if or when cryonics approaches viability. At present, however, the science is so speculative that this does not appear to be a worthwhile use of the public purse. As such, at least at the present time, cryonics appears best left as a private matter, for those optimistic—some would say deluded – few who are willing to make the investment. Shaw suggests that people should be at liberty to make this choice, since their choice only involves a failure of altruism, as it prevents the money being spent on charitable ends and deprives the world of (already scarce) organs available for transplant.^122^

But, as JS’s case illustrates, cryonics can involve public, as well as private, goods. In order to comply with Jackson J’s ruling, the NHS Trust and staff were required to devote resources to effecting JS’s wishes, presumably at some cost to the care of other patients. Maybe this would be less of a concern in a private treatment setting, but that was not the case with JS, so the common interest is evidently engaged.

Assuming that the common interest is engaged, there may be a case for regulation, as some have proposed.^123^ Jackson J acknowledged this, in view of concerns about the practices of the ‘under-equipped and disorganised’ voluntary organization arranging JS’s preservation,^124^ as well as ‘possible public health concerns and the position of the coroner’.^125^ He approved the Trust’s plan to forward information about JS’s case to the Human Tissue Authority,^126^ and wondered if cryonics might be brought within the Authority’s purview ‘if it is to happen in future’.^127^ There may be a case for regulation (for example, setting standards for the relevant organizations) if this could be effected swiftly and inexpensively. But, as with state provision, it can be queried whether regulation is needed at this point in time, as the numbers of actual and potential cryons do not appear to warrant such effort or expense. As Jackson J’s ruling attests, the law arguably already has sufficient tools with which to muddle along, at least until cryonics becomes more widespread.^128^

The second objection, in terms of the common interest, is that cryonics threatens our shared world, and accordingly jeopardizes the resources we share in common. Over-population—and the strains this places on the planet’s resources—is one concern, although Moen argues that the burdens can be alleviated, for example through taxation.^129^ But there is also the environmental burden to consider. This objection is already levelled at traditional forms of disposal, such as cremation and burial, prompting efforts to promote greener alternatives.^130^ Shaw thinks the parallel is unfair because, if it succeeds, cryonics is a form of preserving a life, rather than disposing of the dead.^131^ But that is an inadequate riposte: the fact surely remains that cryopreservation, which might occur over centuries, will be bad for an already ailing world. Presumably Shaw judges the benefits to outweigh the harms, but he tends to see the benefits accruing to the reanimated, and does not explain why their interests should take precedence over the interests of the planet and its other inhabitants, so the objection persists.

### C. Unitary Theories

The case for caution over cryonics gains ground when we turn to *unitary* theories. These see the public interest as consisting in the protection or promotion of objective goods, ‘whereby a person or group’s interests are derived from a theory about what they ideally ought to want or what is good for them, rather than from their subjective preferences’.^132^ On this account, cryonics will be contrary to the public interest if it involves valuing the wrong things. Here, two concerns arise, around the value of death (and the merits of certainty about when death has occurred), and the value of science (and its advancement).

First, cryonics seems to threaten not only the fact *that* we will die (if, that is, it ultimately leads to immorality), but also *when* we can say that someone is dead. There may be value in death; indeed, perhaps we have a duty to die, after having had a ‘fair innings’ of some or other duration.^133^ Whatever the merits of that argument, there will certainly be value in knowing when death has occurred. Cryonics is currently only lawfully available after death, as we currently understand it, has occurred.^134^*Cryon-JS* is therefore dead—but, if the procedure delivers, *reanimated-JS* will be dead no more. This challenges our understanding of death as something irreversible, from which we cannot return. That challenge might be overcome if pre-mortem cryonics—i.e. ‘mercy-freezing’—were to be permitted.^135^ An analogy with life-support seems plausible: the law could signal that the cryon is ‘suspended’ and has not died. Shaw, however, wants to go further and hold that cryopreserving (then reanimating) the deceased is also analogous to life-support.^136^ For this analogy to succeed, we would need to revise our existing definition of death. Both Shaw and Moen appreciate this,^137^ and they favour a concept of ‘information-theoretical death’, i.e. ‘death occurs when the neural structures that encode personality, thoughts, memories, etc, are damaged to such an extent that restoration is in principle impossible’.^138^

Our current definition of death is already under attack, both by those who want to expand the definition (for example, to include as dead those who have lost higher brain function, which might increase the pool of available organ donors),^139^ and by those who seek to restrict it (for example, families who seek continued treatment and care for their brain dead loved ones).^140^ Cryonics presents fresh challenges, since it threatens to destabilize existing definitions and confuse the various *sequelae* of death. Admittedly, the current legal definition of death does permit of evolution, since it is tethered to medical definitions, which change over time.^141^ Cryonics, however, invites more than evolution: it necessitates a radical redefinition. Such redefinition is not unprecedented: the dominant contemporary focus on brain-stem, rather than circulatory, death followed on from developments in organ transplantation.^142^ Despite this, it would be premature to accept cryonics’ invitation at present, given the current state of the science. If the science progresses and the case for redefinition mounts, then various practical and legal arrangements following on from death would also need to be considered. Ettinger, the early pioneer of cryonics, recognized that complex questions would arise, ranging from matrimonial matters (particularly if there are multiple former spouses or civil partners) to financial matters (around estates, life insurance and tax liability).^143^ As Smith says, we need certainty on such matters: ‘Failure to recognise death as death would play havoc not only with the law of property and succession, but act to destabilise the very social and religious fabric of society’.^144^

The second objective value points to medical science and the need for its advancement. The law certainly recognizes this value: despite fears to the contrary,^145^ the courts are already prepared to accommodate innovation, by authorizing experimental procedures in the best interests of patients. For example, in *Simms*,^146^ the High Court approved the use of experimental drugs to treat two incapacitated patients with Creutzfeldt Jakob Disease (vCJD), while in *J*, it authorized a short trial of a sleeping pill, Zolpidem, for a patient in a permanent vegetative state.^147^ Despite such accommodation, the courts are also keen to assert that not just any old (or new) science will make the grade. In the law of negligence, the judges have re-asserted their authority to assess the logical defensibility of the medical opinion they encounter in the courtroom.^148^ The judges have also scrutinized scientific evidence in cases, like JS’s, that involve the welfare of young persons. In *Re C*,^149^ the Court of Appeal upheld a ruling authorizing the immunization of two girls (including with the controversial MMR vaccine), contrary to the wishes of their mothers, with whom they resided, and in line with the wishes of their fathers. Thorpe LJ noted that the rival expert opinions ‘were of unusually unequal force’,^150^ with Sedley LJ going so far as to dismiss the evidence cited on the mothers’ behalf as ‘junk science’.^151^

Although the cases have obvious differences, the precedent in *Re C* potentially offered Jackson J the opportunity to similarly side with a sceptical, estranged father in *Re JS*. Like JS’s father, Jackson J certainly had his misgivings about the evidence for cryonics: ‘The scientific theory underlying cryonics is speculative and controversial’.^152^ However, he chose not to pursue the *Re C* route, and he focused not on the (de)merits of cryonics, but on JS’s interests now, and the comfort she might take from having her wishes honoured. His ruling might nevertheless have offered some tacit support to cryonics. As I noted earlier, by not barring JS’s choice, Jackson J might have nudged open the door towards honouring patients’ demands for unproven—arguably ‘futile’—procedures. Such demands need not be met at present,^153^ although the courts are increasingly seeing futility as a matter to be judged by patients.^154^ Halliday has suggested that the offer of ‘futile’ procedures can serve to promote other values, such as the value of hope.^155^ JS evidently took some comfort from the possibilities of cryonics—but was this cold comfort, offering false hope? Perhaps it is in the public interest to draw a line prohibiting that which can only offer false hope to the vulnerable. If so, cryonics, and by extension Jackson J’s decision, potentially oversteps such a line.

Yet, drawing the line will be difficult: on which side, for example, should the promises of religious or spiritual leaders fall?^156^ Defending too hard a line could also cause science to stagnate, thereby depriving future patients of future innovations, however inconceivable these are presently. Equally, however, the promises of future science should be balanced against the needs of present patients. New developments—in human enhancement, life-extension, personalized medicine and the like—attract attention and, indeed, funding,^157^ but in the here-and-now resources are stretched and everyday healthcare dilemmas—for example, involving ageing populations—persist.^158^ Perhaps these current problems should be prioritized and cryonics de-prioritized, although Shaw would doubtless reply that cryonics is privately funded, so investment therein is at most a failure of altruism. This is persuasive, but more work is undoubtedly needed to ascertain where the balance should be struck between all of the different interests at stake, both present and future.

## VI. CONCLUSION

Writing about cryonics in 1967, Henderson and Ettinger opined: ‘any pessimism whatever, on any score, is grotesquely premature. We have scarcely begun to live and learn’.^159^ Five decades on, reanimation of a cryon still seems a long way off, but cryonicists continue to take the long view: Alcor believes that ‘medical technology will advance further in coming decades than it has in the past several centuries, enabling it to heal damage at the cellular and molecular levels and to restore full physical and mental health’.^160^ Contemporary cryonicists would presumably endorse Henderson and Ettinger’s view that cryonics is ethically defensible:

What parent would hesitate to save a child's life, merely because the child *might* later be unhappy, or *might* later crowd someone? Likewise, in medical ethics, it is the life of the patient that counts, and not the welfare of other individuals or of society.^161^

I have suggested, however, that there are important countervailing considerations, which relate both to the interests of the person undergoing cryonics—JS in our case—and the interests of others, including the wider society and, indeed, the planet.

Starting with the individual seeking cryopreservation, Jackson J rested his decision on JS’s autonomous wish for cryopreservation. As such, he did not let objective considerations about the possible wrongfulness of JS’s choice prevent him from honouring that choice, although his decision can be read as advancing another objective good, in preserving the relationship between JS and her mother. Generally, however, Jackson J followed the dominant legal approach, thereby conceiving of autonomy in subjective terms and finding JS sufficiently intelligent and informed to take the decision to be cryopreserved. Although his reasoning is hard to fault, we can nevertheless ask what it is that entitles and empowers the dying JS to legislate for the future reanimated-JS she might become—assuming, that is, the science can ultimately deliver that which she sought.

Temporal questions also arise if we look at JS’s welfare in a different sense, this time understood in terms of her happiness. Whether or not the dying-JS, the cryon-JS and the reanimated-JS remain identifiably the same person, it is apparent that JS’s interests can differ at different points in time. Some scholars maintain that the reanimated person can be happy and that cryopreservation is a better gamble than certain death. However, their case is not yet complete: relational concerns still linger, particularly around the reanimated person’s family, finances, and fit in the future. Whether or when such future interests should supersede the present interests of the dying individual also remains to be seen. This point was poignantly illustrated in JS’s case, given evidence of her mother’s preoccupation with the cryonics arrangements at the time of JS’s demise.

Finally, it is not only the interests of the prospective cryon, or even those of their close family, that are relevant, since there are public interests at stake. The preponderance view of the public interest—which looks to what the majority of people want—admittedly adds little, since we should not draw firm conclusions about what the majority would support from the mere fact that very few people have elected for cryopreservation. But the common interest—which seeks to fairly promote that which we have in common—does usefully highlight the fact that cryonics appears (unjustly) to be the preserve of the wealthy. In response, the state could seek to make cryonics available to everyone, but the speculative state of the science suggests that state investment would currently be an inappropriate use of public resources. For the same reason, (potentially costly) regulation is probably not yet indicated. Maybe, then, cryonics should remain a private matter, even if the money is better spent on other causes. But, even then, there will still be common interests to consider, not least the environmental burden imposed by cryonics.

There are also relevant objective, unitary interests, concerning the value of death and the value of science. First, cryonics threatens the assumption that death is irreversible and would, if the science advances, require us to revisit the definition of death and the various rules associated with its occurrence. The case for doing so might mount, but is not yet made out. Secondly, there is a balance to be struck between meeting society’s current needs and enabling science to flourish. By deciding in JS’s favour, Jackson J’s decision tacitly favours the latter. Cryonics may yet deliver on JS’s hopes but, as things stand, it might at worst only offer false hope; if so, this is arguably not a position that the law should be endorsing.

In sum, this ruling raises thorny questions about the interests at stake in a decision to undergo cryonics. Further work is invited,^162^ including on the nature of ‘welfare’ and the ‘public interest’, and particularly, on the balance to be struck between current and future interests, and between the public interest and individuals’ interests. As JS’s case demonstrates, these are not merely abstract matters. Hopefully others will take up the challenge of articulating an account of interests that does justice to the different interests at stake, and to the different holders of these interests, both now and in the future.

## Footnotes

1
*Re JS (Disposal of Body)* [2016] EWHC 2859 (Fam), [10].

2 Ibid [23].

3 Eg Jackson J refers to ‘the preservation of sperm and embryos as part of fertility treatment’: *Re JS* (n 1) [7].

4 Like the judge, I will use ‘cryopreservation’ and ‘cryonics’ interchangeably to refer to what JS sought (he also occasionally conflates the two, referring to ‘cryonic preservation’ eg *Re JS* (n 1) [8]).

5 DR Spector, ‘Legal Implications of Cryonics’ (1969) 18 Clev-Marshall L Rev 341.

6 The Immortalist Society, ‘Brief Biography of Robert CW Ettinger’ <http://immortalistsociety.org/robert_ettinger_bio.htm> accessed 5 December 2016. For further detail on the history of the movement, see: AA Perlin, ‘“To Die in Order to Live”: The Need for Legislation Governing Postmortem Cryonic Suspension’ (2007–2008) 36 Sw UL Rev 33.

7 Cryonics Institute <http://www.cryonics.org/> accessed 2 December 2016.

8 G Raynder, L Finnigan and H Bodkin, ‘Girl, 14, who died of Cancer Cryogenically Frozen after Telling Judge she wanted to be brought back to life “in Hundreds of Years”’ The Telegraph (London, 18 November 2016) <http://www.telegraph.co.uk/news/2016/11/18/cancer-girl-14-is-cryogenically-frozen-after-telling-judge-she-w/> accessed 2 December 2016.

9 ALCOR <https://www.alcor.org/> accessed 2 December 2016.

10 Oregon Cryonics <http://www.oregoncryo.com/index.html> accessed 2 December 2016.

11 Trans Time <http://www.transtime.com/index.html> accessed 2 December 2016.

12 KrioRus <http://www.kriorus.com/en/> accessed 2 December 2016.

13 Statis Systems Australia <http://www.stasissystemsaustralia.com/project.html> accessed 2 December 2016.

14 H+Pedia, ‘Cryonics Organisations’ <https://hpluspedia.org/wiki/Category:Cryonics_organisations> accessed 5 December 2016.

15 Society for Venturism, ‘Cryonics Membership’ <http://www.venturist.info/cryonics-membership.html >> accessed 5 December 2016. On the American law pertaining to the protection of this freedom, see: Perlin (n 6) 41–44.

16 Cryonics Institute, ‘Cryonics Procedures’ <http://www.cryonics.org/ci-landing/procedures/> accessed 2 December 2016.

17 Cryonics Institute, ‘Frequently Asked Questions’ <http://www.cryonics.org/about-us/faqs/> accessed 2 December 2016.

18 Ibid.

19
*Re JS* (n 1) [7].

20 Participating as a judge in the ‘Ethical Judgments Project’ reinforced for me the challenges of resolving ethico-legal dilemmas, even in a fictional exercise: SW Smith, J Coggon, C Hobson, R Huxtable, S McGuinness, J Miola, and M Neal, eds, *Ethical Judgments: Re-writing Medical Law* (Hart 2016). I also hope that nothing in this paper contributes further to any distress that JS’s family may be experiencing.

21 GP Smith II, ‘Intimations of Immortality: Clones, Cyrons and the Law’ (1983) 6 UNSW Law J 119, 120.

22
*Re JS* (n 1) [10].

23 Ibid [46].

24 Ibid [7].

25 Ibid [69].

26 Raynder et al (n 8).

27
*Re JS* (n 1) [68].

28 Ibid [23].

29 Smith (n 21) 131; *Donaldson v Van de Kamp* 4 Cal Rptr 2d 59 (Cal Ct App 1992). See also *Alcor Life Extension Foundation, Inc v Mitchell*, 9 Cal Rptr 2d 572, 574 (Ct App 1992), regarding cryonics and the lawful disposal of bodies. For discussion of other US legal activity around cryonics, see: Perlin (n 6).

30
*Re JS* (n 1) [23].

31 The terms are synonymous: *In Re B (A Minor) (Wardship: Sterilisation)* [1988] AC 199, *per* Lord Hailsham LC, 202.

32 s 1(1).

33
*Re JS* (n 1) [46].

34 D Parfit, *Reasons and Persons* (Oxford University Press 1984) 493 (emphasis in original).

35 J Coggon, ‘Varied and Principled Understandings of Autonomy in English Law: Justifiable Inconsistency or Blinkered Moralism?’ (2007) 15 Health Care Anal 235, 241.

36 Ibid.

37 But see ibid.

38 ‘Generally’ because ‘We cannot always have what we want’ (*Aintree v James* [2013] UKSC 67, *per* Lady Hale, [45]).

39 The Law Commission, *Mental Incapacity* (Law Com No 231, HMSO 1995).

40 Contrast *Gillick v West Norfolk and Wisbech Area Health Authority *[1986] AC 112 and Mental Capacity Act 2005, ss 2, 3.

41
*Re R (A Minor) (Wardship: Consent to Treatment)* [1992] Fam 11; *Re W (A Minor) (Consent to Medical Treatment)* [1993] 1 FLR 1.

42 At least, not overtly. See: Coggon (n 35).

43 Law Commission (n 39) [3.4].

44
*Aintree* (n 38) [45].

45
*Re JS* (n 1) [13].

46 Ibid [38], [30] (emphasis in original).

47 Eg *Re B (Adult: Refusal of Medical Treatment)* [2002] 2 All ER 449.

48
*Re JS* (n 1) [46], [31].

49 T Hope, A Slowther and J Eccles, ‘Best Interests, Dementia and the Mental Capacity Act (2005)’ (2009) 35 J Med Ethics 733.

50 Eg R Huxtable, ‘For and Against the Four Principles of Biomedical Ethics’ (2013) 8 Clinical Ethics 39; R Huxtable and R ter Meulen, eds, *The Voices and Rooms of European Bioethics* (Routledge 2015); R de Vries, ‘Bioethics in Academic Rooms: Hearing Other Voices, Living in Other Rooms’, in R Huxtable and R ter Meulen (eds) *The Voices and Rooms of European Bioethics* (Routledge 2015) 192–206, 205.

51 B Lanre-Abass, ‘Autonomy and Interdependence: Quandaries in Research Ethics’ (2012) 4 Health 173, 175.

52 Jackson J refers to JS’s ‘dependence on her mother’ (*Re JS* (n 1) [36]). But see further Section IV, regarding the unfortunate circumstances at the time of JS’s demise.

53
*Gillick* (n 40) *per* Lord Scarman, 186.

54
*Re JS* (n 1) [9].

55 Ibid [25].

56 First, does JS have the competence or capacity to make the decision for cryonics? Jackson J refers to her having ‘the capacity to bring this application’, but capacity and competence are decision-specific: better, then, to state that she has the capacity or competence to decide to undergo cryopreservation. Secondly, is Jackson J using ‘competence’ and ‘capacity’ interchangeably and, if so, is that appropriate? He is not the first judge apparently to do so (eg *Gillick* (n 40)) but ‘competence’, which is determined on the basis of the test articulated in *Gillick* (n 40), applies to those under 16 years of age, while ‘capacity’, which is determined on the basis of the test articulated in the Mental Capacity Act 2005, might best be reserved for those of 16 years and over (see: E Cave, ‘Goodbye Gillick? Identifying and Resolving Problems with the Concept of Child Competence’ (2014) 34 Legal Studies 103, 104–105). If this distinction applies, then as JS was 14 years old at the relevant time, the 2005 Act’s terminology might best be avoided.

57
*Re JS* (n 1) [9].

58 Ibid [6].

59 See eg *HE v A Hospital NHS Trust* [2003] 2 FLR 408.

60 Eg DR Freyer, ‘Care of the Dying Adolescent: Special Considerations’ (2004) 113 Pediatrics 381.

61 The latter point was reiterated by Alcor, in a press release following the ruling: Alcor, ‘Who Decides What We Can Do With Our Body (and Brain)?’ 20 November 2016 <http://www.evidencebasedcryonics.org/2016/11/20/statement/> accessed 2 December 2016.

62 R Huxtable, ‘*Re B (Consent to Treatment: Capacity)*: A Right to Die or Is it Right to Die?’ (2002) 14 Child and Family Law Quarterly 341.

63 Eg *Re B* (n 47).

64
*Re JS* (n 1) [10].

65 See the court’s approach in eg *Re E (A Minor) (Wardship: Medical Treatment)* [1993] 1 FLR 386; *Re M (A Child) (Refusal of Medical Treatment)* [1999] 2 FLR 1097.

66
*Re JS* (n 1) [25].

67 See above (n 41).

68 Cf *Re J (a minor)(wardship: medical treatment)* [1992] 2 FLR 165; *R (on the application of Burke) v General Medical Council* [2005] 3 WLR 1132.

69 Eg JC Youngs. Can the Courts Force the Doctor's Hand? *St George's Healthcare NHS Trust v P* [2015] EWCOP 42. (2016) 24 Med Law Rev 99. Moreover, the decision competes with the rule that no one can dictate what happens to their body after death.

70 An awkward, value-laden, term, but here referring to dying without cryopreservation.

71 L Kutner, ‘The Living Will: A Proposal’ (1969) 44 Indiana Law J 539.

72 R Huxtable, ‘Advance Decisions: Worth the Paper They Are (Not) Written On?’ (2015) 5 BMJ End of Life doi:10.1136/eoljnl-2015-000002.

73 Eg AR Edgar, ‘The Challenge of Transplants to an Intersubjectively Established Sense of Personal Identity’ (2009) 17 Health Care Analysis 123; A Campbell, ‘Why the Body Matters: Reflections on John Harris’s Account of Organ Procurement’ in J Coggon, S Holm, S Chan and T Kushner (eds) *From Reason to Practice in Bioethics: An Anthology Dedicated to the Works of John Harris* (Manchester University Press 2015) 131–141.

74 ‘Apparently’, because studies have suggested that some patients in the vegetative state may be able to follow instructions (A Owen, MR Coleman, M Boly, MH Davis, S Laureys, and JD Pickard, ‘Detecting Awareness in the Vegetative State’ (2006) 313 Science 1402).

75 This might be a ‘transformative experience’ that falls short of identity-interference (L Paul, *Transformative Experience* (OUP 2014) 22). But the degree of psychological difference might mean a new identity is forged; indeed, JS might even become trans-human by being enhanced in some way eg through nanotechnology (OM Moen, ‘The Case for Cryonics’ (2015) J Med Ethics, doi:10.1136/medethics-2015-102715, 2).

76 D Shaw, ‘Cryoethics: Seeking Life After Death’ (2009) 23 Bioethics 515, 521.

77 Moen (n 75) 3.

78
*Re JS* (n 1) [46].

79 Ibid [13].

80 Ibid [36].

81 Moen (n 75) 2, 3.

82 See Shaw (n 76) 520.

83 Shaw (n 76) 516. Of course, JS’s young age is likely to have prevented her from accruing any savings and, indeed, her costs were covered by her grandparents’ efforts to raise the funds.

84 See eg D Sperling, *Posthumous Interests: Legal and Ethical Perspectives* (Cambridge University Press 2008).

85 Moen (n 75) 4.

86 Ibid.

87 Ibid.

88 Shaw (n 76) 517, 519.

89 Moen (n 75) 3.

90 Aristotle, *Nicomachean Ethics*.

91 Thanks to Genevieve Liveley for making this point.

92
*Re JS* (n 1) [68].

93 Note that the Mental Capacity Act 2005 s 4(6) requires reference to the incapacitated patient’s ‘*past and present* wishes and feelings’ (emphasis added) when determining their best interests.

94 I am indebted to Mikey Dunn for this example.

95 R Huxtable, ‘Autonomy, Best Interests and the Public Interest: Treatment, Non-treatment and the Values of Medical Law’ (2014) 22 Medical Law Review 459.

96 Ibid; RE Ashcroft, ‘From Public Interest to Political Justice’ (2004) 13 Camb Q Healthc Ethic 20.

97 Jackson J denies he is setting a precedent (*Re JS* (n 1) [28]). Similar claims have been made before (eg *Re A (Children) (Conjoined Twins: Medical Treatment)* [2001] Fam 147, *per* Ward LJ, 204-205). However, this is a problematic assertion: if a case akin to JS’s were to arise in this jurisdiction, it is difficult to see how the same principles could not apply to the same effect. The ruling might even extend beyond cryonics, given some of the judicial creativity on show. Jackson J manages to avoid (perhaps subvert) existing rules which, for example, prevent minors from appointing executors and prevent patients from dictating their future treatment (although, admittedly, cryopreservation is not strictly ‘treatment’). He also takes a far from literal interpretation of s 62 of the Supreme Court Act 1981. The provision empowers the court ‘to appoint as administrator some person other’ than those who would otherwise have had that power, which in this case would be JS’s parents. Jackson J interprets this as entitling him to remove JS’s father.

98 C Sunstein, ‘On the Expressive Function of Law’ (1996) 144 University of Pennsylvania Law Review 2021.

99
*Thor v Superior Court* (1993) 855 P2d 375 (Supreme Court of California).

100
*Donaldson v Van de Kamp* 4 Cal Rptr 2d 59 (Cal Ct App 1992).

101
*Secretary of State for the Home Department v Robb* [1995] Fam 127.

102 Children Act 1989, s 1(1).

103 For a prominent (albeit outlying) example, see: *Re T (a minor)(wardship: medical treatment)* [1997] 1 All ER 906.

104
*Re JS* (n 1) [12].

105 Ibid [13], [17]–[19]. Jackson J did, however, insist that he was not making orders against third parties (Ibid [27]). Whether that is an accurate depiction is open to question: the judge was explicitly making orders against JS’s father (and his family) and surely, in effect, the ruling also required the compliance of third parties, such as the hospital staff.

106 V Held, *The Public Interest and Individual Interests* (Basic Books 1970).

107 A McHarg, ‘Reconciling Human Rights and the Public Interest: Conceptual Problems and Doctrinal Uncertainty in the Jurisprudence of the European Court of Human Rights’ (1999) 62 Mod LR 671, 674–675.

108 N Bhatia and J Savulescu, ‘Cryonics: Is Freezing Bodies All Hype, Hope, or is it just Logistical and Ethical Hell?’ The Conversation (23 November 2016) <http://www.abc.net.au/news/2016-11-24/cryonics-hype-hope-or-hell/8050278> accessed 5 December 2016.

109 Alcor, ‘Alcor Membership Statistics’ <https://www.alcor.org/AboutAlcor/membershipstats.html> accessed 5 December 2016.

110 Cryonics Institute, ‘Cryonics Institute Member Statistics Details’ <http://www.cryonics.org/ci-landing/member-statistics/> accessed 5 December 2016.

111 KrioRus, <http://www.kriorus.com/en> accessed 5 December 2016.

112 Oregon Cryonics, ‘Cases’ <http://www.oregoncryo.com/caseReports.html> accessed 5 December 2016.

113 J Palomino, ‘Cryonics firm sees market in tech’s quest for eternal youth’ SF Gate (17 July 2015) <http://www.sfgate.com/business/article/Cryonics-firm-sees-market-in-tech-s-quest-for-6391836.php > accessed 5 December 2016.

114 On attitudes to cryonics, see the modest study in: WS Badger, ‘An Exploratory Survey Examining the Familiarity with and Attitudes toward Cryonic Preservation’ (1998) 3 Journal of Evolution and Technology <http://www.jetpress.org/volume3/badger.htm> accessed 4 December 2016. Badger notes that, of those surveyed, men, younger people, and those without religious faith tended to have more positive attitudes towards cryonics than women, those over 25 and theists respectively; women tended to be more concerned about the reanimated person’s future lack of family, as were those who were married (as opposed to single individuals).

115 McHarg (n 107).

116 Perlin (n 6) 58.

117 McHarg (n 107) 676.

118 Ibid 677.

119 Cryonics Institute, ‘Funding for Cryostasis’ <http://www.cryonics.org/ci-landing/funding-for-cryostasis/> accessed 5 December 2016.

120 Eg Alcor offers whole body cryopreservation for $200,000, while head preservation (‘neurocryoperservation’) costs $80,000: Alcor, ‘Alcor Cryopreservation Agreement: Schedule A’ <http://www.alcor.org/BecomeMember/scheduleA.html> accessed 5 December 2016.

121 Shaw (n 76) 516.

122 Ibid 518. This point about altruism and organs raises a different difficulty with the ruling. The ruling effectively overrode the wishes of JS’s father. We might query whether that would have been the outcome, had JS instead been seeking to donate her organs after death. Although, under the Human Tissue Act 2004 s 2(7), only one parental consent would have sufficed, it is known that—in practice—an objection from a relative can operate as a bar on organ harvesting (eg NHS Blood and Transplant, ‘Families saying no to donation results in missed transplant opportunities for UK patients’ (15 January 2016) <http://www.nhsbt.nhs.uk/news-and-media/news-articles/news_2016_01_15.asp > accessed 5 December 2016). Certainly, in a case like JS’s, in which mother and daughter are close, we might hope that her mother’s wishes about donation, in conjunction with her own, would direct the final decision. However, it is conceivable that her father’s scepticism might be heeded. This suggests something of a tension: an altruistic bequest of organs is capable of being overridden by a loved one, while a more self-interested decision to be cryopreserved was not. Thanks to Genevieve Liveley for suggesting that I explore this apparent tension.

123 See eg Spector (n 5) 357; Smith (n 21); Bhatia and Savulescu (n 108).

124
*Re JS* (n 1) [68].

125 Ibid [15].

126 Ibid [69].

127 Ibid.

128 Cf GP Smith II, ‘The IcePerson Cometh: Cryonics, Law and Medicine’ (1983) 1 Health Matrix 23, 31. It is possible that a tipping point will be reached at which the need for specific regulation would outweigh the costs of taking such action. That point might be reached, for example, if the legal floodgates are opened and future claims mount up, to which there are inadequate existing legal answers. For his part, Jackson J noted that future cases like JS’s might arise, but he felt that the courts were equipped to filter out groundless actions (*Re J* (n 1) [37]).

129 Moen (n 75) 4.

130 Shaw (n 76) 518.

131 Ibid.

132 McHarg (n 107) 675.

133 Bhatia and Savulescu (n 108). Cf J Hardwig, ‘Is There a Duty to Die?’ (1997) 27 The Hastings Center Report 34.

134 See eg Alcor, ‘Frequently Asked Questions: Can Alcor Cryopreserve me Prior to Legal Death?’ <https://www.alcor.org/FAQs/faq06.html#death> accessed 5 December 2016; *Donaldson v Van de Kamp* 4 Cal Rptr 2d 59 (Cal Ct App 1992); JP Laboufft, ‘“He Wants To Do What?” Cryonics: Issues in Questionable Medicine and Self-Determination’ (1992) 8 Computer & High Technology Law Journal 469; RW Pommer III, ‘Donaldson v Van de Kamp: Cryonics, Assisted Suicide, and the Challenges of Medical Science’ (1993) 9 Journal of Contemporary Health Law & Policy 589. Cf *R (on the application of Pretty) v DPP* [2002] 1 FLR 268; *R (on the application of Nicklinson and another) v Ministry of Justice; R (on the application of AM) v The Director of Public Prosecutions* [2014] UKSC 38.

135 C Henderson and RCW Ettinger, ‘Cryonic Suspension and the Law’ (1967–1968) 15 UCLA Law Review 414, 418.

136 Shaw (n 76) 517.

137 See also C Cohen, ‘Bioethicists must Rethink the Concept of Death: The Idea of Brain Death is not Appropriate for Cryopreservation’ (2012) 67 Clinics 93.

138 Moen (n 75) 1–2.

139 Eg P Singer, *Rethinking Life and Death: The Collapse of Our Traditional Ethics* (St Martin’s Press 1994).

140 Eg LO Gostin, ‘Legal and Ethical Responsibilities Following Brain Death: The McMath and Muñoz Cases’ (2014) 311 JAMA 903; JE Troyer, ‘Defining Personhood to Death’, in L Hagger and S Woods (eds) *A Good Death? Law and Ethics in Practice* (Ashgate 2013) 41–54.

141 See *Re A (A Minor)* [1992] 3 Med LR 303. The current medical guidance is: Academy of Medical Royal Colleges, *A Code of Practice for the Diagnosis and Confirmation of Death* (Academy of Medical Royal Colleges 2008).

142 See eg RM Sade, ‘Brain Death, Cardiac Death, and the Dead Donor Rule’ (2011) 107 Journal of the South Carolina Medical Association 146.

143 Henderson and Ettinger (n 135). See also Spector (n 5).

144 Smith (n 21) 130.

145 See: J Miola, ‘Bye-bye Bolitho? The Curious Case of the Medical Innovation Bill’ (2015) Medical Law International doi:10.1177/0968533215605667.

146
*Simms v Simms and An NHS Trust* [2002] EWHC 2734.

147
*B NHS Trust v J* [2006] EWHC 3152.

148
*Bolitho v City and Hackney Health Authority* [1998] AC 232.

149
*Re C (A Child)(Immunisation: Parental Rights)* [2003] EWCA Civ 1148.

150 Ibid [24].

151 Ibid [36].

152
*Re JS* (n 1) [7]; cf [30], [31].

153 See (n 68) and the recent, widely-publicised, case of Charlie Gard: *Re Gard (A Child)* [2017] EWHC 1909 (Fam).

154 See eg *Aintree* (n 38).

155 R Halliday, ‘Medical Futility and the Social Context’ (1997) 23 *Journal of Medical Ethics* 148.

156 Bhatia and Savulescu (n 108).

157 Eg CA Gericke, A Riesberg and R Busse, ‘Ethical Issues in Funding Orphan Drug Research and Development’ (2005) 31 J Med Ethics 164.

158 Eg G Birchley, K Jones, R Huxtable, J Dixon, J Kitzinger and L Clare, ‘Dying Well with Reduced Agency: A Scoping Review and Thematic Synthesis of the Decision-Making Process in Dementia, Traumatic Brain Injury and Frailty’ (2016) 17 BMC Medical Ethics, doi:** **10.1186/s12910-016-0129-x.

159 Henderson and Ettinger (n 135) 419.

160 Alcor <http://www.alcor.org/> accessed 2 December 2016.

161 Henderson and Ettinger (n 135) 419.

162 Cf Moen (n 75) 1.

